# TRPM2 Promotes Atherosclerotic Progression in a Mouse Model of Atherosclerosis

**DOI:** 10.3390/cells11091423

**Published:** 2022-04-22

**Authors:** Yunting Zhang, Fan Ying, Xiaoyu Tian, Zhenchuan Lei, Xiao Li, Chun-Yin Lo, Jingxuan Li, Liwen Jiang, Xiaoqiang Yao

**Affiliations:** 1 School of Biomedical Sciences, Heart and Vascular Institute, Li Ka Shing Institute of Health Science, Faculty of Medicine, The Chinese University of Hong Kong, Hong Kong, China; yunting@link.cuhk.edu.hk (Y.Z.); sarayf130@gmail.com (F.Y.); xytian@cuhk.edu.hk (X.T.); 1155160294@link.cuhk.edu.hk (Z.L.); 18826235169@163.com (X.L.); lcy_lo@cuhk.edu.hk (C.-Y.L.); jingxuanli@link.cuhk.edu.hk (J.L.); 2 Centre for Cell and Developmental Biology, State Key Laboratory of Agrobiotechnology, School of Life Sciences, The Chinese University of Hong Kong, Hong Kong, China; ljiang@cuhk.edu.hk

**Keywords:** TRPM2, atherosclerosis, ROS

## Abstract

Atherosclerosis is a chronic inflammatory arterial disease characterized by build-up of atheromatous plaque, which narrows the lumen of arteries. Hypercholesterolemia and excessive oxidative stress in arterial walls are among the main causative factors of atherosclerosis. Transient receptor potential channel M2 (TRPM2) is a Ca^2+^-permeable cation channel activated by oxidative stress. However, the role of TRPM2 in atherosclerosis in animal models is not well studied. In the present study, with the use of adeno-associated virus (AAV)-PCSK9 and TRPM2 knockout (*TRPM2^−/−^*) mice, we determined the role of TRPM2 in hypercholesterolemia-induced atherosclerosis. Our results demonstrated that TRPM2 knockout reduced atherosclerotic plaque area in analysis of En face Oil Red O staining of both whole aortas and aortic-root thin sections. Furthermore, TRPM2 knockout reduced the expression of CD68, α-SMA, and PCNA in the plaque region, suggesting a role of TRPM2 in promoting macrophage infiltration and smooth-muscle cell migration into the lesion area. Moreover, TRPM2 knockout reduced the expression of ICAM-1, MCP-1, and TNFα and decreased the ROS level in the plaque region, suggesting a role of TRPM2 in enhancing monocyte adhesion and promoting vascular inflammation. In bone-marrow-derived macrophages and primary cultured arterial endothelial cells, TRPM2 knockout reduced the production of inflammatory cytokines/factors and decreased ROS production. In addition, a TRPM2 antagonist N-(p-amylcinnamoyl) anthranilic acid (ACA) was able to inhibit atherosclerotic development in an *ApoE^−/−^* mouse model of atherosclerosis. Taken together, the findings of our study demonstrated that TRPM2 contributes to the progression of hypercholesterolemia-induced atherosclerosis. Mechanistically, TRPM2 channels may provide an essential link that can connect ROS to Ca^2+^ and inflammation, consequently promoting atherosclerotic progression.

## 1. Introduction

Atherosclerosis is a chronic inflammatory arterial disease that leads to various complications such as myocardial infarction, stroke, embolization, ulceration, thrombosis, and aneurysm, many of which are important cause of morbidity and mortality [1]. In atherosclerosis, the lumen of arteries narrows due to the build-up of atheromatous plaque. Hypercholesterolemia and excessive oxidative stress in arterial walls are among the main causative factors of atherosclerosis [2,3]. Under oxidative stress, plasma low-density lipoprotein (LDL) is oxidatively modified to form oxidized LDL (oxLDL) in the subendothelial space, where it attracts leukocytes, such as monocytes, to vascular walls [3]. The monocytes then differentiate into macrophages, which subsequently turn into foam cells after taking up oxLDL. These inflammatory macrophages produce excessive reactive oxygen species (ROS) to stimulate multiple pathological events, including inflammation, vascular cell death, vascular wall hyperplasia, and narrowing/occlusion of the blood vessel lumen [3].

In addition to macrophages, dysfunctional vascular endothelial cells and vascular smooth-muscle cells also release excessive ROS to participate in inflammatory responses during atherosclerotic progression. Moreover, during atherosclerotic development, vascular smooth-muscle cells transform from a quiescent contractile phenotype to alternative phenotypes, including proliferative synthetic phenotypes and macrophage-like and foam-cell-like phenotypes [4]. These transformed vascular smooth cells migrate from the media layer to the plaque region, contributing to the development of atherosclerotic plaques [4]. However, atherosclerotic development is a highly complicated process of which many details are still unknown.

Transient receptor potential channel M2 (TRPM2) is a nonselective cation channel permeable to Ca^2+^, Na^+,^ and K^+^. The channel is activated by reactive oxygen species (ROS), adenosine 5′-diphosphoribose (ADP-ribose), and Ca^2+^ [5]. TRPM2 is suggested to be a cellular sensor for oxidative stress [5]. H_2_O_2_, as an endogenous ROS, mainly acts through ADP-ribose to stimulate TRPM2, causing extracellular Ca^2+^ entry and intracellular store Ca^2+^ release [5]. Activity of TRPM2 elicits proinflammatory responses in multiple tissues [6,7,8,9]. Indeed, TRPM2 is recognized as a potential therapeutic target for oxidative-stress-related and inflammation-related pathologies, such as Alzheimer’s disease [10,11] and inflammatory bowel disease [12].

In vascular tissue, TRPM2 is expressed in macrophages, vascular endothelial cells, and vascular smooth-muscle cells [5,13]. Excessive activity of TRPM2 triggers multiple pathophysiological events that are associated with atherosclerotic development, including endothelial barrier dysfunction [14], apoptotic vascular cell death [15,16], and vascular wall hyperplasia [17]. However, despite such circumstantial evidence implying that TRPM2 could play a role in atherosclerosis, the role of TRPM2 in atherosclerosis is still not well studied in animal models.

In the present study, we explored the possible role of TRPM2 in atherosclerosis. Adeno-associated virus (AAV)-mediated overexpression of PCSK9 [18] was used to induce hypercholesterolemia to elicit atherosclerotic development in *TRPM2^+/+^* and *TRPM2^−/−^* mice. The results demonstrated that TRPM2 knockout reduced the atherosclerotic progression. Furthermore, a TRPM2 antagonist N-(p-amylcinnamoyl)anthranilic acid (*ACA*) also inhibited atherosclerotic progression. Bioinformatics study also showed an elevated expression of TRPM2 in atherosclerotic arteries of patients when compared to intact arteries from the same patients.

## 2. Materials and Methods

### 2.1. Experimental Animals

Animals were supplied by the University Laboratory Animal Services Centre and their use was approved by the Ethical Committee of Animal Research (CUHK). The animals used in the present study included apolipoprotein E-deficient (*ApoE^−/−^*) mice, TRPM2 wild-type (*TRPM2^+/+^*), and TRPM2 knockout mice (*TRPM2^−/−^*). The latter two mouse lines were a gift from Yasue Mori Group in Kyoto University, Japan [12]. In *TRPM2^−/−^* mice, the trpm2 gene was disrupted by deleting the exon that contributes to the putative pore region of the TRPM2. The mice were of C57BL/6J background [12]. Only male mice were used in all in vivo studies. The animals were kept at a constant temperature (21 ± 1 °C) under a 12/12 h light/dark cycle and had free access to water and standard chow unless specified.

### 2.2. Primary Cell Culture of Bone-Marrow-Derived Macrophages, Arterial Endothelial Cells, and Arterial Smooth-Muscle Cells

Bone-marrow cells were harvested from the femur and tibia of 6–7-week-old male *TRPM2^+/+^* and *TRPM2^−/−^* mice by sterile PBS flushing with a 23G needle and 10 mL syringe. Bone-marrow cells were passed through a 70 μm cell strainer, then separated by Ficoll-Paque PLUS (GE Healthcare, Uppsala, Sweden). The mononuclear cells in the middle layer were collected, then cultured with M-CSF (10 ng/mL) in RPMI 1640 medium supplemented with 10% fetal bovine serum for seven days to obtain bone-marrow-derived macrophages.

Arterial endothelial cells were isolated from carotid arteries of 4–5-week-old, age-matched male *TRPM2^+/+^* and *TRPM2^−/−^* mice. Carotid arteries were dissected in sterile PBS, followed by incubation with collagenase type IV (Sigma-Aldrich Chemical, St Louis, MO, USA) at 37 °C for 2 h. The detached endothelial cells were collected by centrifugation at 1300 rpm, re-suspended, and cultured in a T25 flask containing complete endothelial-cell growth medium EGM (Lonza Walkersville, Salisbury, MD, USA) supplemented with 10% FBS. After attachment of ~45 min, unattached non-endothelial cells were washed away, and new complete growth medium was added. Seven days later, confluent cells were propagated into one T75 flask. The primary cultured endothelial cells within three passages were used for in vitro assays.

For isolation of arterial smooth-muscle cells, aortas were dissected from male *TRPM2^+/+^* and *TRPM2^−/−^* mice (8–9 weeks of age), and the adventitial layer and endothelial layer were removed. The tissues were cut into pieces, and digested in 10 mg/mL collagen type I (Sigma-Aldrich Chemical, St Louis, MO, USA) at 37 °C for 6–8 h with stirring until the tissues were fully digested. The isolated smooth-muscle cells were cultured with Dulbecco’s Modified Eagle medium containing 10% fetal bovine serum and a combination of penicillin–streptomycin at 37 °C in the room air atmosphere.

### 2.3. Cytosolic Ca^2+^ ([Ca^2+^]_i_) Measurement

Bone-marrow-derived macrophages, primary arterial endothelial cells, and primary arterial smooth-muscle cells were incubated in the dark with 10 µM Fluo-4 (Invitrogen, Eugene, OR, USA) and 0.02% Pluronic F-127 in Ca^2+^-free physiological saline solution (0Ca^2+^-PSS) at 37 °C for 30 min. The cells were bathed in 0Ca^2+^-PSS, then challenged with 500 μM H_2_O_2_ to induce intracellular Ca^2+^ release, followed by 2 mM Ca^2+^ add-back. 0Ca^2+^-PSS contained in mM: 140 NaCl, 5 KCl, 1 MgCl_2_, 10 glucose, 0.2 EGTA, 5 HEPES, pH 7.4. Fluorescence intensity and signal relative to the starting signal (F1/F0 ratio) were measured using an Olympus FV1000 confocal microscope.

### 2.4. Establishment of Atherosclerotic Model

*TRPM2^+/+^* and *TRPM2^−/−^* mice at the age of 8–10 weeks were intravenously injected with AAV-PCSK9 (4 × 10^11^ vg per mouse, WZ Biosciences Inc., Jinan, China). Two different feeding periods of high-cholesterol diet were used for atherosclerotic development, namely two months and four months. For En face Oil Red O analysis of whole aortas, *TRPM2^+/+^*, *TRPM2^−/−^* and *ApoE^−/−^* mice were fed with a high-cholesterol diet (Rodent diet with 45% of calories from carbohydrate, 35% of calories from fat, and 12.5% cholesterol; D12336; Research Diets, New Brunswick, NJ, USA) for four months. The four-month feeding of a high-cholesterol diet led to severe atherosclerosis with total atherosclerotic region close to 40–50%, which made it especially suitable for En face Oil Red O staining assessment of whole aortas. However, the four-month feeding of a high-cholesterol diet led to excessive lesions in the aortic-root area, which made it very difficult to analyze the aortic-root thin section due to a signal-saturation problem. Therefore, for analysis of thin-tissue sections of aortic roots, the mice were fed for two months with a high-cholesterol diet.

### 2.5. Serum Lipid Profile

Blood of *ApoE^−/−^*, *TRPM2^+/+^* and *TRPM2^−/−^* mice was collected via the celiac vein, and serum was obtained by centrifugation at 2000× *g* at room temperature for 10 min. The serum lipid profile was evaluated by a commercially available assay kit (Stanbio, Boerne, TX, USA) specialized for serum total cholesterol (TC), and measured on a plate reader (Bio-Rad, Hercules, CA, USA) by detecting the absorbance at 500 nm.

### 2.6. En Face Oil Red O Staining of Whole Aortas

*ApoE^−/−^*, *TRPM2^+/+^* and *TRPM2^−/−^* mice were killed by CO_2_ asphyxiation. Mouse aortas were dissected in cold PBS and cut open to expose the atherosclerotic plaques. After fixation in 4% formaldehyde for 10 min at 4 °C, the tissues were first rinsed in water for 10 min and then in 60% isopropanol. The aortas were stained with Oil Red O for 20 min with gentle shaking, rinsed in 60% isopropanol, and then rinsed three times in water. The samples were flattened on the glass slides with the endothelial surface facing upwards. The images were recorded using a SONY RX100VI Camera (SONY, Hong Kong, China). The plaque areas were analyzed using National Institutes of Health ImageJ 1.52a software (Rasband, W.S., ImageJ, Bethesda, MD, USA) and calculated by expressing the plaque area relative to the total vascular area.

### 2.7. Histological Examination

Aortic roots in heart tissue were dissected and frozen. For the examination of atherosclerotic lesions in the aortic roots, frozen sections of 8 µm thickness were prepared starting from the three valve cusps of the aortic sinus. The sections were stained with hematoxylin and eosin (H&E), Oil Red O, and Masson’s trichrome. Pictures were taken under the microscope, followed by quantification of the atherosclerosis lesion area in the aortic root using National Institutes of Health ImageJ software. For H&E analysis, manual tracing of the entire intima lesion area and the area of vessel lumen was performed. The relative lesion area was obtained by calculating the ratio of the lesion area versus the area of the vessel lumen. The necrotic core area was also measured. For Oil Red O, the lesion area with positive staining was analyzed.

### 2.8. Immunohistochemical Staining

Frozen sections of 8 µm thickness were prepared from aortic roots. The sections were fixed in 4% formaldehyde, incubated with 3% H_2_O_2_ for 10 min_,_ washed with tris-buffered saline (TBS) plus 0.025% Triton X-100 for 5 min twice with gentle agitation, and blocked with 5% bovine serum albumin in TBS for 30 min at room temperature. The samples were incubated with diluted primary antibody overnight at 4 °C, followed by biotin-conjugated secondary antibody for 1 h at room temperature and then streptavidin-HRP for 1 h at room temperature. Chromogen was developed at room temperature. Pictures were taken under the microscope.

The antibodies used for immunostaining included anti-TRPM2 antibody TM2E3 (1:100; homemade) [15,17], anti-CD68 (1:300; 28058-1-AP; Proteintech, Wuhan, China), anti-α-SMA (1:300; 14395-1-AP; Proteintech), anti-PCNA (1:100; 10205-2-AP; Proteintech,), anti-ICAM-1(1:200; sc-107; Santa Cruz, Dallas, TX, USA), anti-MCP-1 (1:100; 66272-1-Ig; Proteintech), and anti-TNFα (1:100; 17590-1-AP; Proteintech). Quantification of immune-positive signals in the media and neointimal regions but without the adventitial area was performed using Image Pro Plus. The data were expressed as integrated optical density (IOD) divided by area.

### 2.9. Quantitative RT-PCR (qRT-PCR) of Inflammatory Cytokines/Factors

RNA was extracted by using TRIzol Reagent (Thermo) according to the manufacturer’s protocol. cDNA was synthesized using a High-Capacity cDNA Reverse Transcription Kit (Thermo). qRT-PCR was performed using SYBR Select (Thermo Scientific, Hong Kong, China) following the manufacturer’s protocol. β-actin was used as the internal control. Primers used for quantitative PCR were: TNFα-forward 5′-GGTGCCTATGTCTCAGCCTCTT-3′; TNFα-reverse 5′-GCCATAGAACTGATGAGAGGGAG-3′; IL-1β-forward 5′-TGGACCTTCCAGGATGAGGAC-3′; IL-1β-reverse 5′-GTTCATCTCGGAGCCTGTAGTG-3′; IL-6-forward 5′-TACCACTTCACAAGTCGGAGGC-3′; IL-6-reverse 5′-CTGCAAGTGCATCATCGTTGTTC-3′; β-actin-forward 5′-CCTGAGCGCAAGTACTCTGTGT-3′; β-actin-reverse 5′-GCTGATCCACATCTGCTGGAA-3′; CD68-forward 5′-CCCAAGGAACAGAGGAAG-3′; CD68-reverse 5′-GTGGCAGGGTTATGAGTG-3′; aSMA-forward 5′-CCCAGACATCAGGGAGTAATGG-3′; aSMA-reverse 5′- TCTATCGGATACTTCAGCGTCA3′; PCNA-forward 5′- TGCTCTGAGGTACCTGAACT-3′; PCNA-reverse 5′- TGCTTCCTC ATCTTCAATCT-3′; ICAM1- forward 5′- CAATTTCTCATGCCGCACAG-3′; ICAM1-reverse 5′- AGCTGGAAGATCGAAAGTCCG-3′; MCP1-forward 5′- CCCAATGAGTAGGCTGGAGA-3′; MCP1-reverse 5′- AAAATGGATCCACACCTTGC-3′; IL18-forward 5′- ACTGTACAACCGCAGTAATACGC-3′; IL18-reverse 5′- AGTGAACATTACAGATTTATCCC-3′; CRP-forward 5′- AGCCTCTCTCATGCTTTTGG-3′; and CRP-reverse 5′- TGTCTCTTGGTGGCATACGA-3′.

### 2.10. ROS Staining

Frozen sections of 8 µm thickness were prepared from aortic roots, stained with freshly prepared dihydroethidium (DHE) staining solution (5 μM, Beyotime Biotechnology, s0063, Shanghai, China) for 30 min at 37 °C in the dark, washed with PBS and mounted onto glass slides. DHE fluorescent images were taken by a FV1200 confocal microscope with 543 nm of excitation wavelength and analyzed using ImageJ. Similar protocol was used for DHE fluorescence staining of cultured smooth-muscle cells and macrophages.

### 2.11. ACA Administration in Mice

For En face Oil Red O staining of whole aortas, ApoE*^−/−^* mice were fed with a high-cholesterol diet for four months to develop atherosclerosis. In the last three months, the mice were subcutaneously injected once every three days with 25 mg/kg/day ACA or with PBS as control. For examination of thin-tissue sections of aortic roots, ApoE*^−/−^* mice were fed with a high-cholesterol diet for two months to develop atherosclerosis. In the last month, the mice were subcutaneously injected once every three days with 25 mg/kg/day ACA or with PBS as control.

### 2.12. Bioinformatic Analysis

RNA-Seq data were sourced from GSE43292 series of GEO database. The study was conducted from pieces of carotid endarterectomy collected from 32 patients. They were paired, including for each patient one sample of the atheroma plaque (stage IV and over of the Stary classification) containing core and shoulders of the plaque, and one sample of distant macroscopically intact tissue (stages I and II). The samples contained media and neo-intima without adventitia.

### 2.13. Statistical Analysis

For comparison between two groups, analysis was done by unpaired two-tailed Student’s *t*-test. Differences among three or more groups were examined by one-way analysis of variance (ANOVA) test followed by Tukey’s multiple comparisons test. All statistical analysis and calculations were performed using Prism version 5 (GraphPad Software, San Diego, CA, USA). *p* values < 0.05 were considered statistically significant. Data are represented as mean ± standard deviation (SD) or mean ± standard error of the mean (SEM). The number of biological replicates is indicated in individual figures.

## 3. Results

### 3.1. TRPM2 Mediates H_2_O_2_-Stimulated Extracellular Ca^2+^ Entry and Intracellular Store Ca^2+^ Release in Bone-Marrow-Derived Macrophages, Primary Arterial Endothelial Cells, and Primary Arterial Smooth-Muscle Cells

**Figure 1:**
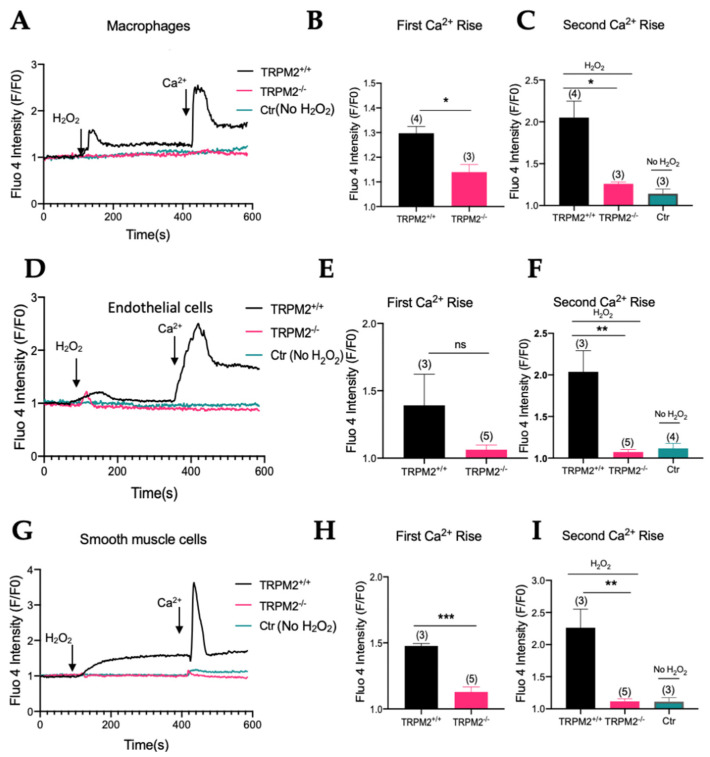
TRPM2 mediates H_2_O_2_-stimulated extracellular Ca^2+^ entry and intracellular Ca^2+^ release in bone-marrow-derived macrophages, primary arterial endothelial cells, and primary arterial smooth-muscle cells. (**A**–**C**) Bone-marrow-derived macrophages from *TRPM2^+/+^* and *TRPM2^−/−^* mice. (**D**–**F**) Primary arterial endothelial cells from *TRPM2^+/+^* and *TRPM2^−/−^* mice. (**G**–**I**) Primary aortic smooth-muscle cells from *TRPM2^+/+^* and *TRPM2^−/−^* mice. The cells were bathed in Ca^2+^-free physiological saline, challenged by 500 µM H_2_O_2_, which elicited the first cytosolic Ca^2+^ rise. Then, 2 mM Ca^2+^ was added-back to initiate the second Ca^2+^ rise. Shown are representative time course (**A**,**D**,**G**) and data summary for the maximal cytosolic Ca^2+^ changes in response to H_2_O_2_ (**B**,**E**,**H**) and Ca^2+^ add-back (**C**,**F**,**I**). Controls were *TRPM2^+/+^* cells without H_2_O_2_ treatment. Mean ± SEM (*n* = 3–5). *, *p* < 0.05; **, *p* < 0.01; ***, *p* < 0.001; ns, not significant.

Bone-marrow-derived macrophages, arterial endothelial cells, and arterial smooth-muscle cells were isolated from *TRPM2^+/+^* and *TRPM2^−/−^* mice. The cells were challenged with H_2_O_2_ to elicit cytosolic Ca^2+^ rise. In order to differentiate H_2_O_2_-stimulated extracellular Ca^2+^ entry from H_2_O_2_-stimulated Ca^2+^ release from intracellular stores, the cells were first bathed in a Ca^2+^-free solution (0Ca^2+^-PSS). Application of H_2_O_2_ at 500 µM initiated a cytosolic Ca^2+^ rise, which presumably was due to H_2_O_2_-stimulated Ca^2+^ store release (Figure 1A,D,G). Then, 2 mM Ca^2+^ was added-back to extracellular bath, causing the second cytosolic Ca^2+^ rise, which was due to Ca^2+^ entry (Figure 1A,D,G). As a control, some cells were bathed in Ca^2+^-free solution without H_2_O_2_ pretreatment; addition of extracellular Ca^2+^ to these cells only induced a very small or no cytosolic Ca^2+^ rise (Figure 1A,D,G). Therefore, the second Ca^2+^ rise in H_2_O_2_-pretreated cells represented the H_2_O_2_-stimulated Ca^2+^ entry. Importantly, H_2_O_2_-stimulated Ca^2+^ entry (Figure 1C,F,I) was substantially reduced in the bone-marrow-derived macrophages, primary-cultured arterial endothelial cells, and primary-cultured arterial smooth-muscle cells from *TRPM2^−/−^* mice when compared with the corresponding cells from *TRPM2^+/+^* mice. H_2_O_2_-stimulated intracellular Ca^2+^ release was also reduced in the bone-marrow-derived macrophages and primary-cultured arterial smooth-muscle cells from *TRPM2^−/−^* mice (Figure 1B,H). These data confirmed a major contribution of TRPM2 in H_2_O_2_-stimulated Ca^2+^ entry and H_2_O_2_-stimulated intracellular Ca^2+^ release in these cell types and indicated the presence of functional TRPM2 in these cells.

### 3.2. Establishment of Mouse Model of Atherosclerosis and Detection of TRPM2 Expression in Atherosclerotic Plaques

To establish a mouse model of atherosclerosis, *TRPM2^+/+^* and *TRPM2^−/−^* mice were infected with AAV-PCSK9 and fed with a high-cholesterol diet. As expected, the serum cholesterol level of these mice infected with AAV-PCSK9 and fed with a high-cholesterol diet was substantially higher than that of control mice without AAV-PCSK9 infection and fed with normal diet (652 ± 114 (*n* = 8) mg/dl for the former group vs. 122 ± 5 (*n* = 5) mg/dl for the latter group, *p* < 0.001). However, there was no difference in serum cholesterol level between TRPM2*^−/−^* and TRPM2^+/+^ mice when both were treated with AAV-PCSK9 and fed with a high-cholesterol diet (652 ± 114 (*n* = 8) mg/dl for the former group vs. 709 ± 225 (*n* = 8) mg/dL for the latter group).

**Figure 2:**
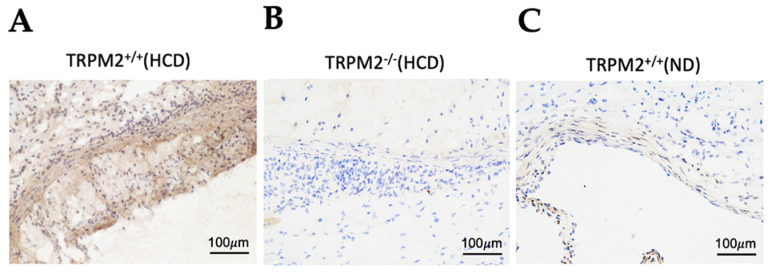
Representative tissue-section images showing TRPM2 protein expression in the plaque regions of aortic roots from high-cholesterol-diet-fed *TRPM2^+/+^* mice (**A**) but not in *TRPM^−/−^* mice (**B**). Tissue-section staining of aortic roots from normal diet (ND) fed *TRPM2^+/+^* mice without atherosclerotic plaques is also shown in (**C**). Brown-color signals represent TRPM2 expression using anti-TRPM2 antibody TM2E3. Blue color represent hematoxylin counterstaining of cell nuclei. Scale bar, 100 µm. For all experiments, shown are representative images from four mice.

Aortic roots were cut into frozen thin sections and subjected to immunohistochemical staining of TRPM2. The expression of TRPM2 could be detected in the atherosclerotic lesion area of *TRPM2^+/+^* mice (Figure 2A) but not in the corresponding region of *TRPM2^−/−^* mice (Figure 2B). In addition, TRPM2 expression could barely be detected in normal vascular tissue of non-atherosclerotic *TRPM2^+/+^* mice (Figure 2C). These data demonstrated that TRPM2 expression was elevated in the atherosclerotic plaque region.

### 3.3. TRPM2 Knockout Ameliorates the Development of Atherosclerotic Plaques in Whole Aortas

**Figure 3:**
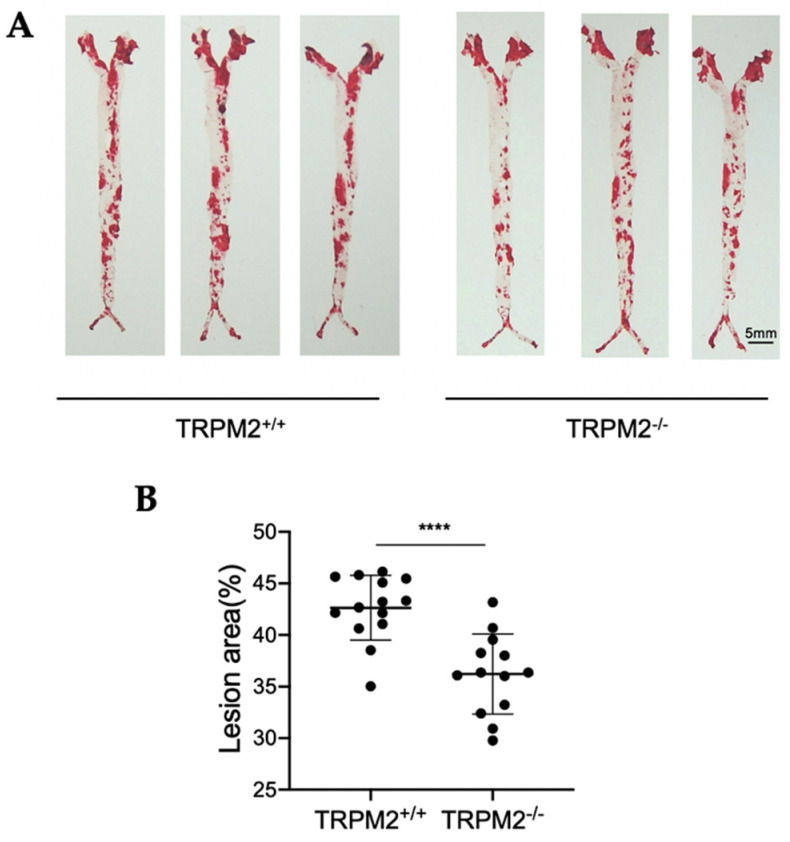
TRPM2 knockout ameliorates the development of atherosclerotic plaques in whole aortas. *TRPM2^+/+^* and *TRPM2^−/−^* mice were injected with AAV-PCSK9 and fed with a high-cholesterol diet for four months. The aortas were dissected, split longitudinally, and pinned open for surface lesion measurements with Oil Red O staining. The atherosclerotic lesion area is visualized as red in (**A**). The lesion area for individual arteries is quantified using Image J and summarized in (**B**). Data are shown as mean ± SD (*n* = 13–14) with each dot representing the data from a single animal. ****, *p* < 0.0001.

En face Oil Red O staining of whole aortas was used to give an overall assessment of the role of TRPM2 channels in atherosclerotic development. *TRPM2^+/+^* and *TRPM2^−/−^* mice were infected with AAV-PCSK9 and fed with a high-cholesterol diet for four months. Whole aortas were dissected out and then subjected to En face Oil Red O staining. The results showed that the atherosclerotic plaques were mostly localized in the aortic arch and around the branched regions of the aorta (Figure 3). Importantly, significant reduction in the atherosclerotic plaque area was observed in *TRPM2^−/−^* mice when compared with that of *TRPM2^+/+^* mice (Figure 3), indicating that knockout of TRPM2 reduced atherosclerotic development.

### 3.4. TRPM2 Knockout Reduces Atherosclerotic Plaques in Aortic Roots

**Figure 4:**
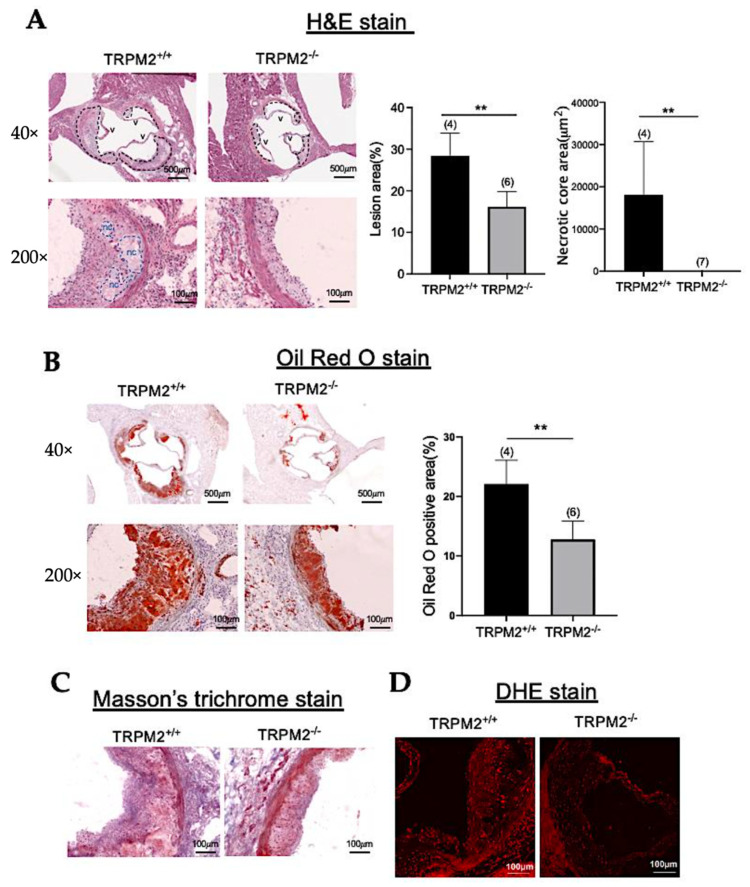
TRPM2 knockout reduces atherosclerotic plaque area in tissue sections of aortic roots. (**A**) Representative tissue-section images (left, with low and high magnification) and data summary (right) of H&E-stained aortic roots from *TRPM2^+/+^* and *TRPM2^−/−^* mice fed with a high-cholesterol diet for two months. Atherosclerotic lesions (outlined with black dashes) and acellular necrotic core (nc) (outlined with blue dashes) are indicated in the images and quantified in bar charts on the right. v stands for aortic valves. (**B**) Representative tissue-section images (left, with low and high magnification) and data summary (right) of aortic roots stained with Oil Red O. Shown are lipid-rich plaques (red) in the aortic roots of *TRPM2^+/+^* and *TRPM2^−/−^* mice. (**C**) Representative high magnification tissue-section images of aortic roots stained with Masson’s trichrome. Collagen is stained in blue. (**D**) Representative images of DHE-stained thin sections of aortic roots from *TRPM2^+/+^* and *TRPM2^−/−^* mice. For all experiments, shown are representative images from four to seven mice. Scale bar, 100 µm or 500 µm as indicated. Summary data are shown as mean ± SD (*n* = 4–7). **, *p* < 0.01.

To explore mechanistic details of TRPM2 in atherosclerosis, frozen thin sections were prepared from aortic roots and then subjected to staining with atherosclerotic markers. Here, *TRPM2^+/+^* and *TRPM2^−/−^* mice were infected with AAV-PCSK9 and fed with a high-cholesterol diet for two months. Frozen thin sections were prepared from aortic roots, and then subjected to hematoxylin and eosin (H&E) staining, Masson’s trichrome staining, and Oil Red O staining. As indicated by H&E staining, TRPM2 knockout reduced the atherosclerotic lesion area and necrotic core area in aortic-root thin-tissue sections (Figure 4A). Oil Red O staining results showed that lipid accumulation in the atherosclerotic lesion area was reduced in *TRPM2^−/−^* mice compared with that in *TRPM2^+/+^* mice (Figure 4B). Masson’s trichrome staining demonstrated that fibrosis level was decreased in the aortic-root thin-tissue sections of *TRPM2^−/−^* mice (Figure 4C). DHE staining of aortic-root sections indicated that ROS production was also reduced in the atherosclerotic lesion area of *TRPM2^−/−^* mice when compared with that of *TRPM2^+/+^* mice (Figure 4D).

### 3.5. TRPM2 Knockout Reduces the Expression of CD68, α-SMA, and PCNA in Plaque Region of Aortic Roots

**Figure 5:**
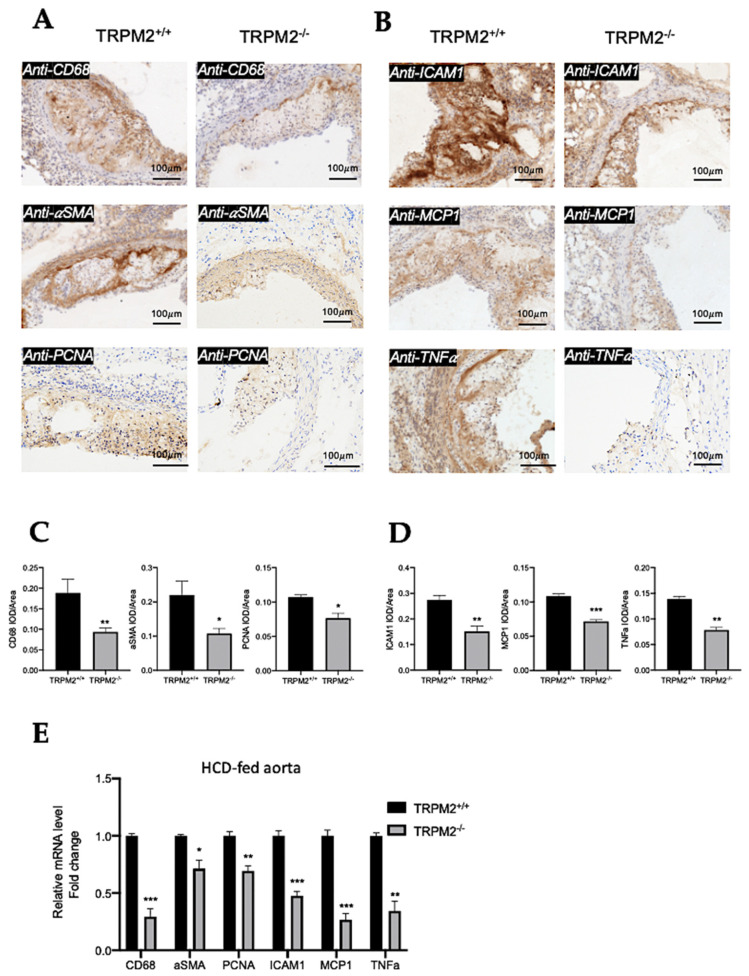
TRPM2 knockout reduces the expression of multiple atherosclerosis-related proteins in the plaque region of aortic roots. (**A**) Representative tissue-section images of immunohistochemical stains of CD68 (upper), α-SMA (middle), and PCNA (lower) in aortic roots from *TRPM2^+/+^* and *TRPM2^−/−^* mice fed with a high-cholesterol diet for two months. (**B**) Representative tissue-section images of immunohistochemical stains of ICAM-1 (upper), MCP-1 (middle), and TNFα (lower) in aortic roots from *TRPM2^+/+^* and *TRPM2^−/−^* mice fed with a high-cholesterol diet for two months. Brown color represents immune-positive signals in lesion area. Blue color represents hematoxylin counterstaining of cell nuclei. For all experiments, shown are representative images from four mice. Scale bar, 100 µm. (**C**,**D**) Quantification of immune-positive signals in (**A**,**B**), expressed as integrated optical density (IOD) divided by area. Mean ± SD (*n* = 4). (**E**) qRT-PCR quantification for the expression of CD68, α-SMA, PCNA, ICAM-1, MCP-1, and TNFα in whole-aorta samples in atherosclerotic mice fed with a high cholesterol-diet for two months. Mean ± SEM (*n* = 3). *, *p* < 0.05; **, *p* < 0.01; ***, *p* < 0.001.

CD68 immunoreactive signals were used to detect infiltrated macrophages and foam cells, whereas α-SMA immunoreactive signals were used to detect transformed vascular smooth-muscle cells in the plaque region of aortic roots. Immunostaining in thin-tissue sections shows that CD68-positive and α-SMA-positive signals were reduced in the plaque area in *TRPM2^−/−^* mice when compared with those in *TRPM2^+/+^* mice (Figure 5A,C). The immunoreactive signals to proliferating cell nuclear antigen (PCNA), a proliferating cell marker, were also reduced in the lesion area of *TRPM2^−/−^* mice when compared with that of *TRPM2^+/+^* mice (Figure 5A,C).

qRT-PCRs were performed to verify the effect of TRPM2 knockout on the expression of CD68, α-SMA, and PCNA in whole-aorta samples prepared from atherosclerotic mice. The results confirmed that the mRNA levels of CD68, α-SMA, and PCNA were lower in the samples from *TRPM2^−/−^* mice than those from *TRPM2^+/+^* mice (Figure 5E).

### 3.6. TRPM2 Knockout Reduces the Expression of ICAM-1, MCP-1, and TNFα in Plaque Region of Aortic Roots

Intercellular adhesion molecule 1 (ICAM-1) and monocyte chemoattractant protein-1 (MCP-1) are important monocyte adhesion molecules and chemokines that can regulate migration and infiltration of monocytes/macrophages. Immunostaining in thin-tissue sections showed decreased expression of ICAM-1 and MCP-1 in the aortic-root plaque area of *TRPM2^−/−^* mice when compared with that of *TRPM2^+/+^* mice (Figure 5B,D). Tumor necrosis factor alpha (TNFα), an inflammatory cytokine, was selected to monitor inflammation. The results showed that TRPM2 knockout reduced the expression of TNFα in the plaque area (Figure 5B,D).

qRT-PCRs were performed to verify the effect of TRPM2 knockout on the expression of ICAM-1, MCP-1, and TNFα in whole-aorta samples prepared from atherosclerotic mice. The results confirmed that the mRNA levels of ICAM-1, MCP-1, and TNFα were lower in the samples from *TRPM2^−/−^* mice than in those from *TRPM2^+/+^* mice (Figure 5E).

### 3.7. TRPM2 Knockout Reduces the Production of Inflammatory Cytokines and ROS in Bone-Marrow-Derived Macrophages and Primary Cultured Vascular Cells

**Figure 6:**
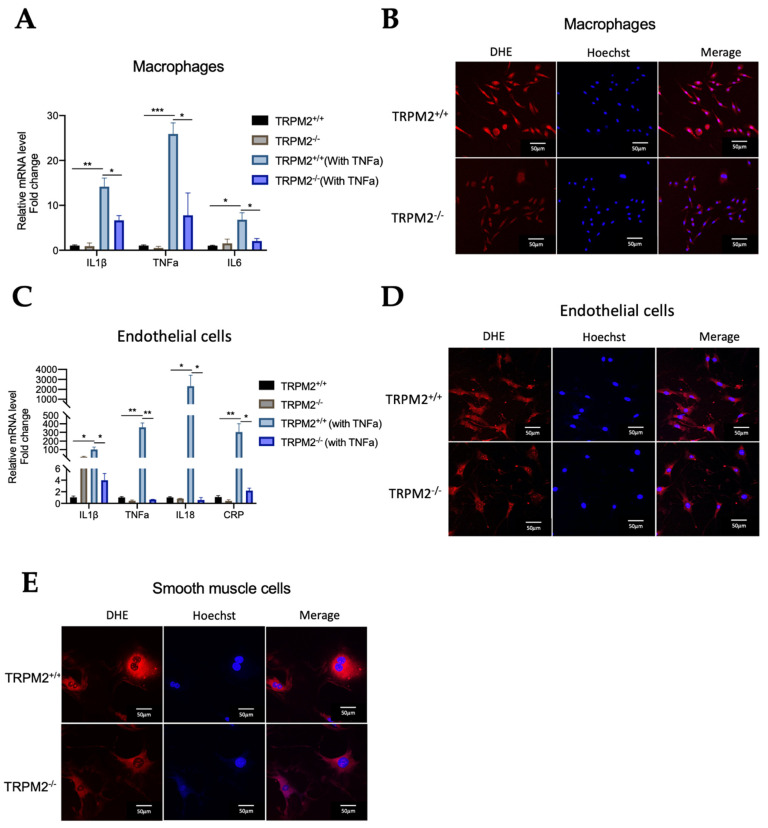
TRPM2 knockout reduces the production of inflammatory cytokines and ROS in bone-marrow-derived macrophages and primary vascular cells. (**A**,**C**) qRT-PCR quantification for the production of inflammatory cytokines/factors in TNFα-treated bone-marrow-derived macrophages (**A**) and primary arterial endothelial cells (**C**). Data from the cells without TNFα stimulation were normalized to 1. Mean ± SEM (*n* = 3–5), * *p* < 0.05, ** *p* < 0.01, *** *p* < 0.001. (**B**,**D**,**E**), Representative images of DHE-stained bone-marrow-derived macrophages (**B**), primary arterial endothelial cells (**D**), and primary arterial smooth- muscle cells (**E**) from *TRPM2^+/+^* and *TRPM2^−/−^* mice. DHE stains are shown in red while DAPI nuclear counterstain is shown in blue. For all experiments, shown are representative images from three mice. Scale bar, 50 µm.

To further study the role of TRPM2 in inflammation, bone-marrow-derived macrophages were isolated and treated with 10 ng/mL TNFα for 4 h to stimulate production of inflammatory cytokines. qRT-PCR results showed that TNFα-induced production of inflammatory cytokines, including IL-1β, TNFα, and IL6, was markedly reduced in the macrophages from *TRPM2^−/−^* mice when compared with that from *TRPM2^+/+^* mice (Figure 6A). Similarly, in the primary cultured arterial endothelial cells, TNFα-induced production of inflammatory cytokines/factors, including IL-1β, TNFα, IL-18, and c-reactive proteins (CRP), was also markedly reduced in *TRPM2^−/−^* mice when compared with *TRPM2^+/+^* mice (Figure 6C).

The cell-permeable ROS-sensitive fluorescent dye DHE was used to measure ROS levels in the primary macrophages, arterial endothelial cells, and arterial smooth-muscle cells. The cells were treated with 10 ng/mL TNFα for 4 h to stimulate ROS production. The results showed that ROS production was much higher in the cells from *TRPM2^+/+^* mice than corresponding cells from *TRPM2^−/−^* mice (Figure 6B,D,E).

### 3.8. ACA Inhibits Atherosclerotic Progression in ApoE^−/−^ Mouse Model of Atherosclerosis

**Figure 7:**
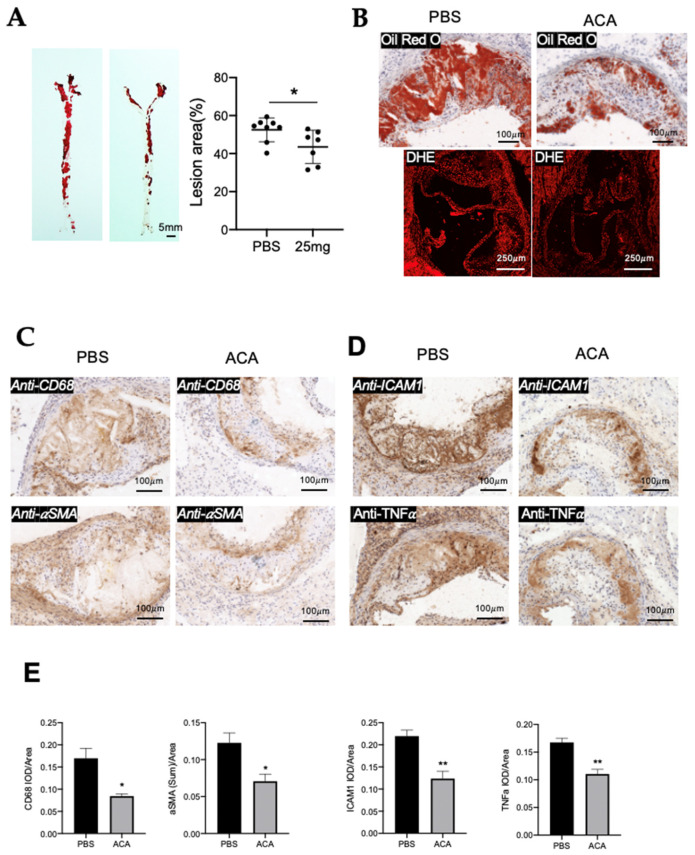

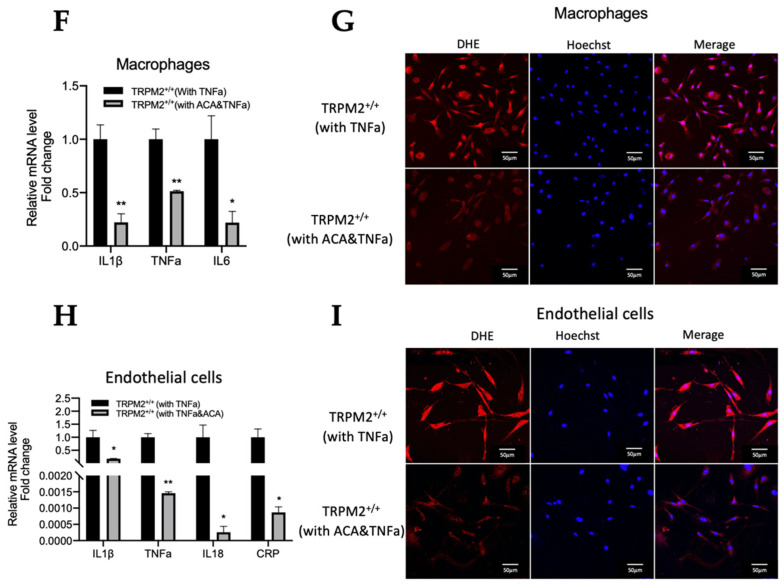
ACA inhibits atherosclerotic progression in an *ApoE^−/−^* mouse model of atherosclerosis. (**A**) En face Oil Red O staining of whole aorta showed that ACA treatment once every three days with 25 mg/kg/day reduced the atherosclerotic lesion area in the whole aorta. Shown are representative images (left) and data summary (right). Mean ± SD (*n* = 7–8) with each dot representing the data from a single animal. *, *p* < 0.05. (**B**–**D**) Effect of ACA treatment on atherosclerotic-lesion-related indexes in tissue sections of aortic roots. (**B**) Representative images of Oil Red O staining (upper) and DHE staining (lower) in the plaque region of thin-tissue sections. (**C**) Representative immunostaining images for CD68 and α-SMA in aortic-root plaque region of thin-tissue section. (**D**) Representative immunostaining images for ICAM-1 and TNFα in aortic-root plaque region. For (**B**–**D**), shown are representative images from four mice. (**E**) Quantification of immune-positive signals in (**C**,**D**) expressed as integrated optical density (IOD) divided by area. Mean ± SD (*n* = 4). (**F**,**H**) qRT-PCR quantification for TNFα-induced production of inflammatory cytokines/factors in bone-marrow-derived macrophages (**F**) and primary arterial endothelial cells (**H**). Mean ± SEM (*n* = 3–6). *, *p* < 0.05; **, *p* < 0.01. (**G**,**I**) Representative images of DHE-stained bone-marrow-derived macrophages (**G**) and primary arterial endothelial cells (**I**) from *TRPM2^+/+^* and *TRPM2^−/−^* mice. DHE stains are shown in red while DAPI nuclear counterstain is shown in blue. For all experiments, shown are representative images from three mice. Scale bar, 50 µm.

We determined the effect of a TRPM2 antagonist ACA on atherosclerotic progression in an *ApoE^−/−^* mouse model of atherosclerosis. En face Oil Red O results showed that, compared with the control mice injected with PBS, injection of ACA at 25 mg/kg/day significantly reduced the atherosclerotic plaque area of the whole-aorta preparation (Figure 7A).

We also examined thin-tissue sections of aortic roots. The results showed that, compared with the control injected with PBS, injection of ACA at 25 mg/kg/day reduced the lipid accumulation and ROS production in aortic roots (Figure 7B). ACA treatment also reduced the expression of atherosclerosis-related proteins, including CD68, α-SMA, ICAM-1, and TNFα, in the plaque area (Figure 7C–E).

We next determined the effect of 25 µM ACA on TNFα-induced production of inflammatory cytokines/factors and ROS in primary cultured cells, including bone-marrow-derived macrophages and primary arterial endothelial cells. ACA treatment clearly reduced the TNFα-induced production of IL-1β, TNFα, and IL6 in macrophages (Figure 7F), and it also reduced the TNFα-induced production of IL-1β, TNFα, IL-18, and c-reactive proteins in endothelial cells (Figure 7H). Furthermore, 25 µM ACA also reduced TNFα-induced production of ROS in macrophages (Figure 7G) and endothelial cells (Figure 7I).

### 3.9. TRPM2 mRNA Expression Is Elevated in Atherosclerotic Region of Patients

**Figure 8:**
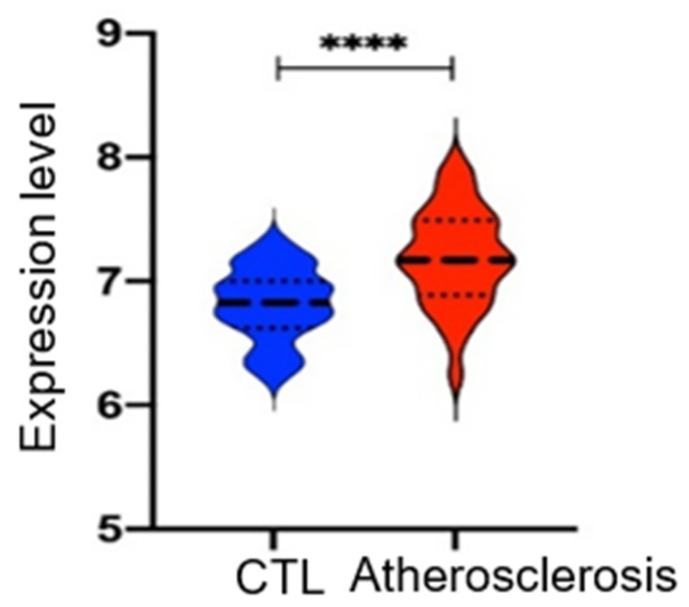
TRPM2 mRNA expression is elevated in atherosclerotic regions of carotid arteries when compared to that in intact regions from the same patients. Bioinformatics analysis was performed with RNA-Seq data from GSE43292 series of GEO database which contain paired samples from 32 atherosclerotic patients. Shown are violin plots with mean and quartiles, *n* = 32. Atherosclerosis indicates atherosclerotic regions; CTL indicates intact regions. **** *p* < 0.0001.

To increase the clinical relevance of the study, bioinformatics analysis was performed to compare the expressional levels of TRPM2 between atherosclerotic carotid arteries and intact arteries in a database of 32 atherosclerotic patients. The results demonstrated that TRPM2 mRNA expression is elevated in atherosclerotic regions of carotid arteries when compared to normal intact regions in these patients’ samples (Figure 8).

## 4. Discussion

Previous studies from us and others have linked TRPM2 to a variety of different cellular processes that are associated with atherosclerotic progression [14,15,16,19]. For example, TRPM2 channels promote vascular cell death via inducing cellular Ca^2+^ overload [15] and/or promoting excessive autolysosomal degradation [16]. TRPM2 channels also enhance neutrophil migration across the endothelial barrier [14], promote smooth-muscle cell proliferation and migration [17], and contribute to injury-induced vascular wall hyperplasia [17]. However, despite such circumstantial evidence for a role of TRPM2 in atherosclerosis, until now there has been no concrete evidence demonstrating that TRPM2 indeed contributes to atherosclerosis in an animal model. In the present study, with the use of AAV-PCSK9 [18] and *TRPM2^−/−^* mice, we explored the possible role of TRPM2 in hypercholesterolemia-induced atherosclerosis. For atherosclerotic analysis, we used En face Oil Red O staining of mouse whole aortas together with immunostaining analysis of atherosclerotic markers in aortic-root thin-tissue sections. The results from theses analyses showed that knockout of TRPM2 markedly reduced the atherosclerotic lesion area in a mouse model of atherosclerosis. Our study provided concrete evidence that TRPM2 indeed contributes to the progression of hypercholesterolemia-induced atherosclerosis in an AAV-PCSK9 mouse model.

ROS and Ca^2+^ homeostasis are two key determinants in atherosclerotic development. ROS are released from macrophages, neutrophils, and vascular cells [3]. It is well documented that excessive production of ROS promotes atherosclerotic progression by stimulating multiple pathological events, including the phenotypic switch of vascular smooth-muscle cells and the migration of these cells into lesion areas, monocyte/macrophage infiltration into lesion areas, inflammation, and cell death [3,20]. Intriguingly, dysregulation of Ca^2+^ homeostasis in vascular cells could also trigger similar pathological events [21,22]. Herein, we hypothesize that TRPM2 channels may provide an essential link that can connect ROS to Ca^2+^ during atherosclerotic development. In this scheme, ROS activates TRPM2 channels to induce cytosolic Ca^2+^ rise in macrophages and vascular cells [12,15,16,23]. This cytosolic Ca^2+^ rise may in turn further stimulate ROS overproduction in these cells [22,24], forming a vicious cycle of ROS overproduction and abnormal Ca^2+^ signaling. Therefore, TRPM2 is a key player in this vicious cycle of ROS overproduction and abnormal cytosolic Ca^2+^ rise in atherosclerosis progression. Supporting this, we found that compared to *TRPM2^+/+^* mice, *TRPM2^−/−^* mice displayed a reduced ROS production in the atherosclerotic lesion area in vivo, and a reduced ROS production in bone-marrow-derived macrophages, arterial endothelial cells, and arterial smooth-muscle cells in vitro. We also found that H_2_O_2_ as an ROS could stimulate Ca^2+^ entry in macrophages, endothelial cells, and smooth-muscle cells, the effect of which was reduced in *TRPM2^−/−^* mice. Moreover, the expressional levels of α-SMA and CD68 were reduced in the plaque area of *TRPM2^−/−^* mice compared with those of *TRPM2^+/+^* mice, implying that TRPM2 may also promote macrophage infiltration and smooth muscle migration into lesion areas. These data provide mechanistic insights about the role of TRPM2 in atherosclerotic development.

Inflammation is another hallmark in atherosclerotic plaque [3,25]. During atherosclerotic development, excessive ROS stimulate proinflammatory macrophages to release inflammatory cytokines, such as TNF-a, IL-1b, IL-6, and other proinflammatory molecules including adhesion molecules (such as ICAM-1) and chemotactic molecules (such as MCP-1), consequently promoting inflammatory responses and aggravating atherosclerotic progression [25]. Herein, we hypothesize that TRPM2 may provide a crucial intermediate link between ROS and inflammatory responses. A likely scenario is that excessive ROS activate TRPM2 to enhance the release of proinflammatory molecules from macrophages and vascular cells, consequently promoting atherosclerosis. Supporting this, we found that knockout of TRPM2 substantially reduced the expression of TNF-a, ICAM-1, and MCP-1 in atherosclerotic plaque areas in vivo. Furthermore, in vitro studies with primary cultured cells demonstrated that knockout of TRPM2 reduced the production of TNF-a, IL-1b, and IL-6 in the macrophages, and reduced the production of IL-1β, TNFα, IL-18, and CRP in arterial endothelial cells.

The critical importance of TRPM2 in multiple processes of atherosclerosis prompted us to explore the possibility of using TRPM2 antagonists as anti-atherosclerotic agents. N-(p-amylcinnamoyl) anthranilic acid (ACA) has been widely used as a TRPM2 inhibitor with IC_50_ of ~1.7 µM. Therefore, we tested its action in atherosclerosis and found that administration of ACA once every three days at 25 mg/kg/day significantly reduced the atherosclerotic lesion area as indicated by En face Oil Red O staining of whole aortas. Further analysis of aortic-root thin-tissue sections also confirmed the anti-atherosclerotic action of ACA, based on Oil Red O staining, immunoreactivity to CD68, α-SMA, ICAM-1, TNFα, and DHE-based ROS staining. Moreover, ACA treatment reduced the production of inflammatory cytokines/factors in primary macrophages and arterial endothelial cells. These results highlight an intriguing possibility of developing TRPM2 inhibitors as potential anti-atherosclerotic agents. Note that although ACA has previously been used in an animal model in vivo to study the involvement of TRPM2 in ischemic brain damage [26], its safety profile has not been extensively studied. Further study of its safety profile is needed for future development of ACA as an anti-atherosclerotic agent. Another point to note that ACA is not very specific to TRPM2. In addition to its inhibitory effect on TRPM2, ACA also inhibits phospholipase A2 [27] and several other TRP channels including TRPM8 and TRPC6 [28]. Therefore, we cannot exclude the possibility that the anti-atherosclerotic effect of ACA could be partly due to its inhibitory actions on phospholipase A2 and/or other TRP channels.

## 5. Conclusions

Our present study demonstrated a critical role of TRPM2 channels in promoting hypercholesterolemia-induced atherosclerosis in AAV-PCSK9 mouse. We show that TRPM2 may enhance ROS production, stimulate inflammation, and promote macrophage infiltration into the vascular wall, consequently aggravating atherosclerotic progression. This study highlights the possibility of targeting TRPM2 and/or its signaling pathways as a potential therapeutic target for atherosclerosis.

## Data Availability

The data presented in this study are available on request from the corresponding author.
